# Supplementary material for: Are carbon emissions trading and green financial instruments synergistic? -Comprehensive quantitative research based on content analysis
Source: PLoS One. 2024 Mar 7;19(3):e0298601. doi: 10.1371/journal.pone.0298601 (PMC10919601; doi:10.1371/journal.pone.0298601)
Supplement: S1 Data — (ZIP) [file pone.0298601.s001.zip › Data/地级市Z_F工作报告.zip!/░▓╗╒/╗╞╔╜╩╨/└┤╫╘╣1⁄2╓┌║┼í╛┬φ┐╦╩2╛▌═°í┐_2010.docx]

各位代表:
　　现在，我代表黄山市人民政府，向大会作工作报告，请予审议，并请市政协委员和其他列席人员提出意见。

        一、2009年工作回顾
　　刚刚过去的2009年，是新世纪以来我市经济社会发展最为困难的一年，也是我们以昂扬的精神状态砥砺奋进并取得突出成就的一年。在省委、省政府和市委的坚强领导下，全市上下以开展深入学习实践科学发展观活动为动力，以加快建设现代国际旅游城市为目标，以推进“十大工程”和“四区建设”为抓手，立足于保增长、保民生、保稳定，着眼于打基础、管长远、增后劲，果断实施了应对危机的一系列政策措施，较好地完成了市五届人大四次会议确定的年度目标任务。预计实现生产总值270亿元，同比增长11.5%，超年计划1个百分点；财政收入突破30亿元，达31.2亿元，增长21.4%，高于全省4.4个百分点；城镇居民人均可支配收入14068元、农民人均纯收入5700元，分别增长9.9%和10.5%。全市呈现出整体经济运行平稳、社会事业全面加强、民计民生继续改善、生态文明持续向好的发展态势，我们在推进科学发展上迈出了坚实的步伐，在现代国际旅游城市建设上掀开了崭新的一页。
　　（一）着力扩大三大需求，促进经济平稳较快发展。扎实开展重大项目建设年活动，全面提速“十大工程”重点项目，全年争取新增国债项目476个、资金5.64亿元，完成固定资产投资348.4亿元，同比增长30.6%，其中省“861”行动计划重点项目增幅位列全省第一。综合交通提速升级工程完成投入23.4亿元，全市关注的合福铁路客运专线正式开工，黄杭铁路、皖赣铁路扩能改造前期工作有力推进；国省道路网改造、宣黄高速公路建设步伐加快，黄祁高速公路全线开工，黄山机场升级改造（一期）基本完成。50万伏徽州输变电工程、城市燃气项目开工建设，累计完成水利、电力、燃气、通信设施投入20.29亿元。在重点抓好旅游消费的同时，着力激活城乡消费市场，全年预计实现社会消费品零售总额107.6亿元，增长19%，位居全省前列。房地产市场健康发展，全年商品房销售面积增长77%；认真实施万村千乡市场工程，家电下乡万户购买量全省第一。全面落实外贸促进政策，帮助企业走出去开拓市场，在外需不振的情况下，全市仍完成外贸进出口总额2.34亿美元，增长31%，成为全省唯一进出口全面增长的地市，并跻身中国城市外贸竞争力百强行列。
　　（二）强力推进结构调整，综合经济实力稳步提高。预计三次产业比例调整到13.2∶41.1∶45.7，第二产业上升了1.6个百分点。认真落实强农惠农政策，着力实施“八个一批”，促进了农村经济稳步发展。十大特色产业基地初具规模，新增绿色、有机、无公害农产品认证面积13.7万亩；全市规模以上农产品加工企业实现营销收入42.15亿元，增长21.9%，超亿元龙头企业达到7家；茶叶产量、产值继续保持全省第一，综合产值达35.5亿元，8家企业进入全国茶业百强。按照“四个一批”的要求，加大企业帮扶力度，减轻企业负担1.06亿元，帮助企业落实各类资金44亿元，推动了工业经济提速增效。规上工业实现增加值73.6亿元，增长26.1%，发展速度和利税增幅均居全省前列，是2003年以来综合运行质量最好的一年；全年引进工业项目227个，规上企业净增75户；工业技改投入完成43亿元，永新股份公司被评为国家创新型企业；全市园区建设快速推进，共计完成投资50亿元，新增入园企业180户，市循环经济园建设全面启动。强力推进旅游“三进”营销，积极实施“双十”计划，实现了旅游经济逆势上扬。全市游客接待量达到2122.52万人次，其中接待入境游客87.71万人次，分别增长17.83%和8.23%，预计旅游总收入168.15亿元，创汇2.44亿美元，分别增长19.34%和10.86%，四项指标增幅高于全国平均水平、总量连续四年全省第一；黄山风景区进山人数达到235.62万人，黄山旅游集团经营收入超过14亿元，均创历史新高。
　　（三）全面深化改革开放，经济发展活力显著增强。成功举办纪念邓小平同志“黄山谈话”三十周年、第十三届中国黄山国际旅游节暨徽文化节、世博安徽论坛等一系列重大活动，黄山航空口岸扩大对外国籍飞机开放获得国家批复，省政府批准设立以黄山为中心的皖南国际旅游文化示范区，国家服务业综合改革试点城市争取工作取得积极进展，编号为79316的小行星被命名为“黄山星”。招商引资取得显著成效，积极参与泛长三角区域发展分工合作，认真组织参加第四届中博会、第五届中国国际徽商大会，全年共引进市外资金220亿元、实际利用外资1.44亿美元，同比增长36.5%和11.2%。抓住我省与中央企业新一轮全方位合作的机遇，与中国五矿、港中旅等中央企业签署合作协议，总投资达185亿元。不断扩大对外交往，与美国檀香山市、南非开普敦市达成建立友城关系意向。全面启动医药卫生体制改革，扎实推进政府机构改革、国有资产管理改革、文化体制改革、农村综合改革、集体林权制度改革和村级公益事业建设“一事一议”财政奖补试点。启动黎阳镇等11个省级扩权强镇试点，县域经济发展进一步提速，增幅高于全市1个百分点。列入全省改革规划的66户国有企业完成改制59户。全市非公有制经济增加值占到生产总值的59%，36家企业分别入选全省民营企业营业收入、纳税总额和出口创汇“百强”。超常规推进金融创新发展，金融机构新增贷款66.8亿元，增长36.4%，高出全省2.8个百分点。批准组建3家农村合作银行、1家村镇银行和8家小额贷款公司，新成立金融机构数为历年最多。
　　（四）全力加快城乡建设，基础条件不断完善。统筹推进城乡建设管理提升工程，修、编城市总体规划等24项规划，完成全市城镇建设投资165亿元，城镇化率提高到41.7%。着力抓好屯溪中心城区建设，完成投入60亿元。高标准、快节奏推进新安江延伸段综合开发，累计完成投资5.5亿元，湖边水利枢纽、花山坝部分工程完工。新建主次干道9条，对天都大道等4条主干道实施黑色化改造，敷设供水管道30余公里，建成污水管道13公里。新建商品住宅81万平方米，新增和延长公交线路2条，提升改造亮化12公里，人均公共绿地达到15.46平方米。市城投公司融资12.4亿元，同比增长185.1%，6亿元企业债券发行得到批准。全市区县城建设步伐加快，247个项目完成投资69.9亿元，新建道路23条，新建改建水厂3座、供水和污水管网51.5公里、133.12公里；44个建制镇全部实现垃圾统一焚烧和填埋，有38个实现集中供水，20个特色镇建设有序推开。扎实推进新农村建设巩固拓展工程，投入建设资金12.5亿元，新增“十有”标准达标村、基本达标村各50个，农村公路“村村通”建成769.9公里，农村安全饮用水覆盖率达94.28%，新建乡镇综合文化站41个、农家书屋174个，乡镇卫生院17所、村卫生室95所，573个自然村实现广播电视村村通，49个村建成农民体育健身工程，又有6个乡（镇）、65个村被列入省“千村百镇”示范村、镇，18个乡镇被评为省级、国家级环境优美乡镇，黟县宏村进入首批全国生态文化村行列。
　　（五）认真实施民生工程，人民生活水平继续改善。深入开展“七保”工作，大力实施“春风行动”，扎实推进30项民生工程，共投入资金10亿元，惠及125万城乡居民，解决了一批事关群众切身利益的突出问题。加大就业再就业工作力度，新增城镇就业、下岗失业人员再就业1.99万和1.06万人，转移农村劳动力27.91万人，城镇登记失业率控制在3.8%。着力扩大社会保障覆盖面，全市城镇基本养老、医疗、失业保险参保人数分别达到11.2万、34.32万和10.74万人，按时足额发放企业退休人员养老金4.31亿元，城镇医疗保险覆盖率和新型农村合作医疗参合率分别达到95%、94.35%，被征地农民社会保障制度全面实施。不断完善社会救助体系，发放城镇和农村低保金9333万元，医疗救助金发放首次超过千万元，低保补助水平和救助金发放增幅均居全省前列；新建和改扩建农村五保供养服务机构29所；6550户家庭解决了廉租住房或住房补贴。继续开展整村推进扶贫工程，投入3004万元，实施295个扶贫项目，预计减少贫困人口7000人。全市“一卡通”发放涉农补贴4.02亿元，人均受益344元，为历史最好水平。
　　（六）切实加大公共投入，各项社会事业协调发展。全市科教文卫体支出13.17亿元，同比增长27.9%。举办首届科技成果展示暨科技项目对接会，全市新增7家省级企业技术中心、4家工程技术研究中心，入选国家创新基金项目29项、高新技术产品47个，7项科技成果获省科学技术奖，歙县、休宁县跻身全国科技进步先进县行列，黄山风景区被评为全国首批数字化景区。完善义务教育经费保障机制，实施义务教育学校绩效工资制度，制定出台义务教育均衡发展实施意见，完成中小学校舍安全工程加固改造14.76万平方米，高考本科达线率和普职招生比位居全省前列，市职业技术学院筹建启动。编制完成徽州文化生态保护实验区规划，率先在全省完成非物质文化遗产普查，全面启动“百村千幢”古民居保护利用工程，市美术馆、黄梅戏馆投入使用，大型多媒体歌舞“徽韵”荣获省精神文明建设“五个一工程”奖，屯溪老街改造提升工程稳步推进，跻身首批十大中国历史文化名街行列。与中央电视台联合推出首届中国武术职业联赛，市五运会和国际山地车节、健走节、登山大会以及“黄山论剑”等活动成功举办。城乡卫生服务体系逐步完善，5个县级医疗机构开工建设，甲型H1N1流感等重大传染病防控扎实有力。夯实计划生育基层基础工作，人口自然增长率2.83‰。着力加强生态建设，人工造林5.7万亩，综合治理水土流失面积95平方公里，污水处理实现区县城全覆盖，完成省政府下达的年度节能减排目标任务。启动农村社区建设试点工作，深化城市社区管理体制改革，圆满完成第三届社区居委会换届选举，屯溪区被列为全国和谐社区建设示范城区。全面加强应急管理体系建设，认真开展安全生产年活动，亿元生产总值安全生产事故死亡人数下降13.5%。深入开展信访积案化解年活动，积极排查化解各类矛盾纠纷。不断健全社会治安防控体系，严厉打击各类违法犯罪活动，顺利完成国庆60周年安保各项任务，确保了全市社会政治稳定。歙县、祁门县等6个区县跻身全省平安县（区）行列，黄山管委会等单位被评为全国社会治安综合治理先进集体，我市再次荣获全国社会治安综合治理优秀市称号。国防动员、人民防空、后备力量建设和双拥工作进一步加强，被评为全国征兵工作先进单位。妇女、未成年人、老年人、残疾人权益得到较好保障，新闻出版、文学艺术、哲学社会科学以及统计、档案、地方志、气象、地质、地震和民族、宗教、对台等工作取得新的成绩。
　（七）扎实开展学习实践活动，政府自身建设得到加强。全市政府系统认真开展深入学习实践科学发展观活动，切实加强思想政治建设，深入查摆和整改工作中存在的突出问题，学习实践活动群众满意率和比较满意率达到100%。深化机关效能建设，大力推进电子政务，全面加强政府信息公开和政务公开工作，着力提高行政服务中心办事效率，项目办理平均承诺时限压缩到5个工作日以内，现场办结率达到85%。组织涉企收费清理和减免政策落实情况专项检查，取消、变更收费项目107项。深入推进以作风、行风为重点的“四大整治”，开展“百家企业评机关”和效能、政风、行风建设“三位一体”评议，继续办好“政风行风热线”和“市民连线”，累计受理群众和企业来信来电1.3万余件。将依法行政工作纳入政府年度目标管理考核，加大规范性文件监督管理力度，被确定为省依法行政工作示范市。自觉接受人大及其常委会的法律监督、工作监督和政协的民主监督，政府系统承办的597件人大代表建议和政协提案全部办结。认真落实党风廉政建设责任制，扎实推进惩防体系建设，严格执行中央厉行节约有关规定，切实强化审计监督和行政监察，严肃查处了一批违法违纪案件。
　　总结过去的一年，我们也清醒地认识到存在的矛盾和问题。一是经济又好又快发展的基础还不稳固，总量不大、速度不快、结构不优的问题依然突出，产业投资尤其是工业投资还不足，牵动性强的项目还不多。二是影响社会和谐的问题仍然不少，居民收入和社会保障水平还不高，就业、教育、医疗等一些事关群众切身利益的问题尚未根本解决，安全生产、生态环保和维护稳定等方面的任务还十分繁重。三是政府工作仍有不少薄弱环节，少数工作人员精神不振、干劲不足，作风不实、服务不优，依法行政、廉洁从政与人民的期待、与岗位的要求还存在一定的差距。对此，我们将认真面对，齐心协力加以解决。
　　各位代表，过去一年，我们在危机中抢抓机遇，在困难中寻求突破，在逆势中保持增长，在竞争中彰显特色，成绩令人鼓舞，发展超乎预期，这是省委、省政府和市委总揽全局、正确领导的结果，是市人大、市政协有力监督、大力支持的结果，是全市上下凝心聚力、顽强拼搏的结果。在此，我代表市人民政府，向全市广大工人、农民、知识分子、干部和离退休老同志，向驻黄解放军指战员、武警官兵和政法干警，向各民主党派、各人民团体和各界人士，致以崇高的敬意！向关心、支持黄山现代化建设的中央、省驻黄单位和海内外友好人士，向在我市创业的投资者、建设者，表示衷心的感谢！

　　二、2010年主要任务
　　今年是全面实现“十一五”规划目标、衔接“十二五”发展的关键之年。总体上看，经济发展环境将好于去年，我国经济在全球率先实现回升向好，宏观调控取向“总体稳定、稳中有调”，继续实施积极的财政政策和适度宽松的货币政策，我省也将加快推进包括皖南国际旅游文化示范区在内的区域协调发展战略，这为我们提供了较多的有利条件和发展机遇。中央和省里明确提出“五个更加注重”、“五个着力”的总体要求，强调要把保持经济平稳较快发展和加快经济发展方式转变有机统一起来，寓调结构于稳增长过程、寓稳增长于调结构之中，这为我们指明了下步的工作重点和努力方向。只要我们坚持好字当头、又好又快的发展主旨，充分把握利用各种积极因素和政策措施，清醒认识并周密做好应对区域竞争和困难挑战的准备，抢机遇稳增长、调结构促转变、强优势硬支撑、打基础增后劲、惠民生保稳定，就一定能够在建设现代国际旅游城市上迈出新的更大步伐。
　　根据市委四届十次全会精神，今年政府工作的总体要求是：深入贯彻落实科学发展观，按照党的十七大、十七届三中、四中全会和中央、省经济工作会议的决策部署，紧紧围绕建设现代国际旅游城市的战略目标，紧紧围绕转变经济发展方式的战略任务，以“十大工程”为主要抓手，突出扩大有效需求，突出提升经济结构，突出统筹城乡发展，突出改善民计民生，突出维护社会稳定，加快构建“四大体系”，加快推进“四区建设”，努力实现经济社会平稳较快发展，全面完成“十一五”规划目标任务并为“十二五”发展奠定坚实基础。
　　综合各种因素考虑，今年经济社会发展的主要预期目标是：地区生产总值增长12%以上，财政收入增长16%；城镇居民人均可支配收入增长10%，农民人均纯收入增长9%；社会消费品零售总额增长16%以上，居民消费价格涨幅控制在3%左右；城镇登记失业率控制在3.9%以内；人口自然增长率控制在4.5‰以内；万元生产总值能耗下降4%，主要污染物排放总量控制在省政府下达目标以内。具体执行中，只要是符合科学发展的，能快则快，好中求快。
重点做好以下九个方面工作：
　　（一）抓紧抓好项目建设，切实扩大有效投入
　　坚持以规划带项目、以项目融资金、以招商兴产业、以投资保增长，加快项目建设、扩大投资规模，优化项目结构、提高投资效益，确保全社会固定资产投资增长28%。
　　全力推进重点项目。按照“五个一批”的工作思路，抓好605个“十大工程”和274个省“861”项目建设，力争新上项目全部开工，159个项目竣工运营。落实构建现代基础设施体系实施意见，抓好综合交通提速升级工程，继续推进黄祁高速、宣黄高速、50万伏徽州输变电工程，力促“三条高铁”加快建设。精心组织、科学编制我市“十二五”规划，紧扣国家政策导向和战略性新兴产业布局，谋深谋透一批重大基础设施、现代服务设施、优势产业发展项目，积极主动与国家和省“十二五”规划对接，争取一批重大项目列入规划，全市项目储备超2000亿元。进一步强化项目工作机制，发挥政府投资项目表率作用，提高企业投资项目服务水平，确保项目推进更快更好。
　　全力推进招商引资。发挥园区、企业、小分队的招商主体作用，着力抓好资源招商、以商引商、地块招商、园区招商和瞄准大企业招商，力争在引进优势产业、新兴产业、财源企业等大项目好项目上有新的突破，在祁门钨钼矿勘查开发、黄山风景区旅游深度开发等央企合作项目上有大的进展，全年引进内资突破300亿元，实际利用外资1.6亿美元。进一步营造招商引资氛围，完善政策，优化机制，抓好项目引进前置服务和落地全程代办代理，鼓励多措并举降低商务成本、提高配套水平，形成宜商、惠商、安商的综合环境优势。加快建立全市共享的客商信息库、在谈项目库、推介项目库和招商信息网，提高招商引资的效率和水平。
　　全力推进市场融资。全面落实支持中小企业发展的金融政策，建立中小企业贷款风险补偿基金，加大金融产品创新和放贷激励力度，力争全市贷款增幅高于全省平均水平。办好银企对接会，引导县域金融机构新增贷款主要投向当地。支持企业通过股份制改造引进战略投资者，积极争取国际金融组织和外国政府贷款。组建市担保行业协会，做大做实担保体系。大力引进商业银行在我市建立分支机构，积极发展地方金融组织，新建农村商业银行、村镇银行各1家，新增小额贷款公司3家以上。认真落实鼓励和扶持企业上市政策，加强对23户拟上市企业的培育，确保有3户企业完成股改、2户企业完成辅导，力争尽快上市。
　　（二）推进旅游产业转型升级，积极发展现代服务业
　　深入贯彻国务院加快发展旅游业的意见，抓住国家扩大居民消费需求和我省建设皖南国际旅游文化示范区的机遇，加快推进旅游产业“五个转变”，加快打造具有国际影响力、竞争力的世界级旅游目的地，努力实现质量效益与规模速度的同步增长，力争全年接待游客2500万人次、旅游总收入突破200亿元。
　　抓营销，巩固拓展市场。坚持政府引导、企业主体、各方联合、市场运作，加大宣传策划创意力度，加强产品线路整合包装，努力使我市旅游主题形象更有影响力、更具冲击力。深入主要城市群开展“三进”营销，有力有效撬动旅游市场。组织开展日韩、港澳台等5个以上主要境外客源地的推介促销，力争入境游客突破100万人次。继续办好黄山国际旅游节暨徽文化节、山地车节、登山大会等19项节庆赛事活动，确保季季有活动、月月有亮点。紧扣“中国世博旅游年”主题，发挥列入世博主题体验之旅示范点城市的优势，出台世博旅游营销政策，开展“世博在上海、旅游到黄山”专题营销，力争接待世博游客超500万人次。加强与国家旅游局驻外办事处、四大总社、跨国机构、知名媒体以及周边城市的合作，运用好长三角“15+1”旅游合作机制和古徽州乡村旅游联盟平台，增强市场开拓的合力。
　　抓建设，加快产业升级。围绕发展旅游观光、休闲度假、文化体验、康体养生、节庆赛事、商务会议六大业态，推进“山、江、湖、城、街、点、村”等15个重要节点建设，提升全市46家A级景区品质，89个旅游综合开发提升工程项目完成投资66.5亿元。发挥黄山风景区龙头示范作用，加大国际精品旅游景区建设力度，加快实施西海大峡谷地轨缆车、景区生物多样性保护等10个新建项目和西海饭店整体改造、景区停车场建设等10个续建项目。着力抓好元一大观、醉温泉、徽州府衙、中法乡村旅游合作等项目以及雨润、太平湖、东黄山、奇墅湖等度假区建设。深化旅游体制机制改革，做大做强旅游企业，筹建旅游投资公司，规范调整旅游资源对外出让、承包和收益分配行为，促进重要旅游资源整合开发，推进旅游业规模化、品牌化、网络化发展。修编乡村旅游发展规划，建设乡村旅游专业村135个，发展“农家乐”接待户500户，力争西递、宏村进入5A级景区行列。扶持壮大20家骨干旅游商品生产企业，大力开发地方特色旅游商品。进一步优化旅游环境，加强市场管理，加快完善旅游服务体系、应急体系和诚信体系。推进“数字黄山”“三网一库一热线”和“智慧黄山”框架下的精品旅游信息化建设，强化旅游集散、停车、公交服务功能，实施旅游管理人员和员工队伍培训计划，提高从业者整体素质。
　　抓重点，发展服务产业。全面落实省加快服务业发展若干意见，争创国家服务业综合改革试点城市。发挥旅游的辐射带动作用，加快相关特色优势服务业的融合发展。规划调整布局屯溪中心城区商业街结构，积极引进知名品牌和零售集团，努力培育若干特色商业街区，初步完成屯溪老街三条横街开发和业态提升，拓展城市旅游消费功能。提高土地供应和开发利用效率，加快中低价位、中小套型普通商品住房建设，加强商品房销售管理，促进房地产市场平稳健康发展。进一步优化楼盘品质和居住环境，打造“最佳第二居住地”品牌。大力激活城乡消费，积极实施城市社区“双进工程”、万村千乡市场工程，做好家电和汽车摩托车下乡工作，继续推进屯溪区省级城市商贸服务示范区试点。加快海宁、永佳等物流中心建设，培育大型批发市场和现代物流企业。积极支持保险、证券机构在我市发展业务。
　　（三）做大做强工业经济，加快新型工业化进程
　　坚持把发展工业作为培育财源、增强实力、争先进位的关键来抓，按照“三个围绕一个不上”的原则和“三个集中”的要求，强力推进园区经济提速增效工程，确保规模以上工业增加值增长25%以上。
　　扩张现有企业，培育优势产业。加快落实构建优势产业体系实施意见，着力发展七大优势产业，加大节能环保、新型显示、生物医药、高性能材料、新能源汽车、光伏、文化创意等战略性新兴产业培育引进力度。抓好产业链补缺补强、龙头企业培育培强，支持永佳集团、金马股份、嵊峰袜业、兴乐铜业、黄山工业泵、皖南机床、黄汽电、黄山电器、谢裕大茶业、松萝有机茶、杉森木业等重点骨干企业加快发展，确保规上企业超500户，产值超亿元企业达45户。引导企业大力发展外向型经济，在抓好技术装备、紧缺材料进口的同时，做大重点出口产业，力争8家企业出口超千万美元，外贸进出口总额增长26%。强化对重点行业、重点企业的运行监测和协调服务，帮助企业解决困难和问题。整合各类资金，扩大市工业发展专项资金规模，增强引导功能，提高使用效益。
　　依托八大园区，壮大园区经济。扎实开展园区建设发展推进年和工业招商质量提升年活动，进一步拉开园区框架，推进园区基础设施建设，积极发展生产性服务业，提高园区产业配套和综合承载能力，全市园区新增开发面积10平方公里。编制实施全市园区产业发展总体规划，出台配套政策，引导园区按照定位发展优势特色产业集群，加快建设特色园区和“园中园”。支持徽州区和歙县联手共建市循环经济园，力争进入全省循环经济试点行列。抓好市经济开发区建设，确保新签项目40个，开工、竣工项目各30个。力争全市园区完成投资75亿元，新签项目180个,竣工项目120个，产值、税收分别达到200亿元和7亿元。
　　推进科技创新，抓好企业改革。以实施重点产业调整振兴规划为契机，着力抓好年产240台五轴连动加工中心（机床）、年产1亿只新型电力电子半导体器件、年清洁化生产万吨绿茶等200个投资超千万元重点技改项目，力争完成工业技改投资50亿元，高新技术产业总产值突破80亿元。把科技创新与发展新兴产业、壮大优势产业、提升传统产业结合起来，深化与高校院所产学研合作，加快科技孵化器等创新服务平台建设，鼓励高新技术企业、省级工程技术研究中心加大研发力度，争创国家级企业技术中心。积极实施知识产权战略，大力培育著名商标和名牌产品。开展企业改制攻坚年活动，全面完成市直43户国有集体企业改制工作，妥善解决改制企业遗留问题。
　　（四）扎实做好三农工作，推动农村改革发展
　　按照统筹城乡发展、抓好“八个一批”的要求，认真落实各项强农惠农政策，深入实施新农村建设巩固拓展工程，促进农业增效、农民增收，实现农业农村发展的新突破。
　　着力发展山区现代农业。继续实施农业产业化“1122”计划，做大做强特色产业，新建农业标准化基地3～5个、规模化养殖示范小区20个，发展绿色、有机、无公害农产品认证面积2万亩，新增3个省级以上名牌产品，力争超亿元、超5000万元的龙头企业分别达到8家、18家，农民专业合作社发展到400个以上，其中规范化运作的示范社达到20个。大力实施林业产业化“4646”工程，加快林业的产业化经营和区域化布局，调整林种树种结构，把油茶产业培育成为兴林富民的支柱产业，新建“一竹四果”和花卉苗木产业基地3万亩，全市林业总产值达到46亿元。加快实施生态茶园“双十”工程，建成高效生态茶园2万亩，建设黄山茶叶实物标样系统工程，办好黄山茶叶暨名优农产品交易会等系列推介活动，力争茶业综合产值突破40亿元。加强重大植物病虫害统防统治和动物疫病防控，确保农产品质量安全。
　　着力改善农村生产生活条件。加强农田水利基本建设，完成水利兴修土石方450万立方米，改造机电灌站50座，新建蓄水池500个、抗旱机井1000口，除险加固小型病险水库29座。继续推进农村电网完善工程。加强农村公路建设，完成“村村通”硬化工程150.7公里。进一步完善文化基础设施，大力推进农村信息网建设。建成标准化乡镇卫生院14所、村卫生室95所。加快示范村建设，建成“十有”标准达标村、基本达标村各50个。
　　着力推进农村各项改革。深化农村综合改革，继续推进乡镇机构改革，进一步改善乡镇公共服务，全面开展村级公益事业建设“一事一议”财政奖补试点。稳定和完善农村基本经营制度，按照“三个不得”和依法自愿有偿原则，加快土地承包经营权流转，积极开展土地整治整村推进试点，完成农村宅基地、集体建设用地使用权确权和登记发证工作。进一步做好水稻、油菜、能繁母猪等政策性农业保险试点工作，探索开展政策性森林保险。加快集体林权制度配套改革，完善林业要素市场建设，积极组建林业担保公司，力争林业贷款突破2亿元。
　　（五）加大城镇建设力度，增强综合承载能力
　　黄山市新一轮城市总体规划，为城市建设发展描绘了新的蓝图。规划凸显了市域南部城镇群的重要地位，确立了由屯溪、新城、岩寺三大组团构成市中心城区的基本框架，提出了2015年、2030年分别建成51万、85万人口城市的目标。今年是规划实施起步之年，必须加快推进城乡建设管理提升工程，确保完成城镇投资200亿元，城镇化率提高1.6个百分点以上。
　　突出抓好城镇规划和管理。强化规划龙头地位，高水平编制城乡发展总体规划、重点发展建设区域规划、重要支撑专项规划、城乡环境风貌整治规划等四大类11项规划，加快构建新型城镇规划建设体系。坚决维护规划的严肃性和权威性，认真组织重点项目建设方案的论证比选，不断加大依法控违拆违力度。以争创全国创建文明城市工作先进城市和蝉联省文明城市为目标，推进城市文明提升工程，推行市容精细化管理，推动综合执法体制向乡镇和街道延伸，突出整治背街小巷、城乡结合部等薄弱地段，整治门前三包、占道经营、户外广告、交通秩序、小区物业等薄弱环节，着力解决油烟、噪声、渣土扰民问题。
　　突出抓好屯溪中心城区建设。计划安排建设项目95个，总投资80亿元；其中政府投资项目22个，投资20.4亿元。着力完善老城区、调整开发区、建设新城区，加快“三桥五片九点十路”建设。认真做好项目前期工作，尽早开工华山大桥、汉沙大桥和梅林大桥，建设梅林大道南段、新潭二号路、码头路，贯通新园路、湖边路、新区路，拓宽改造花山旅游公路，完善老城区和东、西两个片区路网。完成翻板坝至花山谜窟新安江两岸工程，打造湖边水利枢纽、航运码头等精品亮点，实现新安江延伸段蓄水通航。抓好5个地块的旧城改造。完善供水、供电、供气、停车、公交、公厕、无障碍等市政公用设施。加大保徽、改徽、建徽力度，出新主干道两侧建筑立面，升级改造部分绿化亮化工程。加大城市建设融资力度，增强市城投公司、国有资产运营公司投融资能力，深化与开行、农发行及其他商业银行的合作，抓好企业债券发行、土地收储出让以及城市资源的资本化运作。集中力量做好征地拆迁工作，为城市建设提供有力保障。
　　突出抓好城镇群、区县城和小城镇建设。加快构建市域南部城镇群，建设齐云大道、屯光大道延伸段，争取启动城北快速通道工程，开展高铁站站前区10平方公里土地平整和主干路网建设，加快开发徽州区纳尼亚小镇、歙县经济开发区二期、休宁县水南板块，形成东西对接、南北联动的发展态势。抓好甘棠城区外环路工程和黟县城东新区、祁门县新城区等基础设施建设，进一步扩大规模、完善功能、提升品位、增强辐射。发挥资源、产业和区位优势，重点抓好谭家桥、太平湖、潜口、万安等20个特色城镇建设和发展，力争全市44个建制镇完成投资43.4亿元。
　　（六）大力弘扬徽州文化，促进文化大发展大繁荣
　　落实建设文化繁荣先行区实施意见，深入推进文化产业精品打造工程，促进文化与旅游发展相结合，力争文化产业增加值占生产总值比重达到7.5%。
　　加大徽文化资源保护开发力度。认真实施徽州文化生态保护实验区建设规划，开展非物质文化遗产名录、文物保护单位申报和文物普查工作。抓好徽文化研究应用，推进徽州文书文献整理编纂出版工程，加快徽学研究保护档案资料数据库、徽州传统技艺人才档案库建设，出版《黄山市志》。全力推进“百村千幢”古民居保护利用工程，确保完成33个古村落和505幢古民居保护与利用工作，重点是抓好湖边古村落、“徽州小镇”等三个屯溪中心城区异地迁移保护项目，着力打造10个具有旅游新业态特色的古村落和50处乡村艺术会所、民俗客栈和特色酒店。
　　加大文化产业发展力度。积极落实文化产业发展政策，编制文化产业发展规划，抓好文化创意园区等32个重大项目建设，扶持壮大15家市级文化产业示范基地，力争有3家晋升省级示范基地行列。积极推进徽文化艺术长廊工程，启动徽州四雕暨非物质文化遗产博览园、徽派盆景产业园项目。以徽州文化博物馆等16个博物馆（园）为依托，发展文博产业。加快推进文房四宝等旅游文化商品的集群式、产业化发展。大力开发影视演艺产品，推出百处最佳旅游摄影点，打响“徽韵”、“黄梅唱黄山”等一批旅游定点演出，推出“徽州六绝”旅游表演项目。积极稳妥推进文化体制改革，着力推进市歌舞剧团、影剧院转企改制工作，积极引进文化产业集团。
　　加大公共文化服务体系建设力度。着力提高城乡公共文化服务体系的覆盖率和达标率。积极筹建市图书馆、档案馆，谋划改建城市规划馆，新建乡镇综合文化站21个、农家书屋165个，完成591个自然村广播电视村村通任务，数字电视用户发展到3万户以上。实施文化市场综合执法，抓好网吧、棋牌室等娱乐场所规范化管理。继续打造广场文化品牌。办好渐江诞辰400周年纪念活动和第四届徽州读书节，力争有20个画展在我市举办。围绕“迎世博、讲文明、树新风”主题，广泛开展“我为黄山添光彩、我与黄山共荣耀”主题教育实践活动。积极发展哲学社会科学、文学艺术、新闻出版事业。开展全民健身运动，推进农民体育健身工程，建成市游泳馆、健身馆并投入使用；积极组团参加省第十二届运动会，争取最好成绩。
　　（七）加强生态文明建设，努力实现可持续发展
　　落实建设生态文明展示区实施意见，推进生态环境保护建设工程，大力发展绿色经济、低碳经济、循环经济，争取在建立绿色低碳的经济体系和消费模式上走在全省前列。
　　切实保护生态环境。加强78处各级各类自然生态资源的保护与管理，继续推进黄山风景区全国生态监察试点。巩固退耕还林、封山育林成果，积极发展碳汇林业，完成人工造林6万亩、生物防火隔离带90公里，加快实施松材线虫病三道防线工程。消灭交通干线两侧宜林荒山和裸露山体，综合治理水土流失面积90平方公里。加强河道管理，依法取缔各类违法采砂点，严禁在饮用水源地建设与保护水源无关的项目，加快渔亭、王村、齐云山、呈坎等地集中供水厂建设，再解决6.8万农村人口饮水安全问题。力争月潭水库项目立项和新安江流域生态补偿机制试点正式启动。争取再创建省级以上环境优美乡镇5个、生态文明村10个。
　　切实抓好节能减排。引导企业加大对节能减排改造项目的投入，确保完成“十一五”节能减排目标任务。严格执行建设项目环评及“三同时”、试生产管理制度，集中查处和整治涉铅、造纸、化工等行业违法排污问题。实施黄山风景区污水处理提升工程，加快汤口、宏村、深渡、北岸等镇污水处理项目前期工作。争取一批村镇列入省农村清洁工程试点，50个乡镇、150个村分别建成垃圾集中处置和收集转运系统，所有区县城垃圾无害化处理设施投入运营。推进市及部分区县天然气利用工程，新建农村户用沼气池2000口。落实省、市节能奖励办法，严格执行建筑节能强制标准，实施旅游节能节水减排工程，加快景观和道路照明节能改造，抓好30户年耗标准煤2000吨以上重点企业的节能降耗。落实建设用地投资强度和容积率“双控”标准，加大批而未用、供而不建土地清理处置力度，推进土地集约节约利用。
　　（八）强力创新体制机制，提高对内对外开放水平
　　立足黄山实际，加大改革开放力度，为又好又快发展提供良好的制度保障和有利的外部环境。
　　扎实推进重点领域和关键环节的改革攻坚，拓展区域合作和外向发展空间。完成市县政府机构改革任务，稳步推进事业单位绩效工资实施工作。深化医药卫生体制改革，扎实开展黄山区、歙县基层医药卫生体制综合改革省级试点。加大财政管理创新力度，提高财政科学化精细化管理水平。在抓好财源培植、税收征管，提高收入质量、增加可用财力的同时，进一步加强支出管理，优化支出结构，深入推进国库集中支付、政法经费保障和公务消费、公务接待等制度改革，集中更多的财力保障和改善民生，投向经济社会发展重点领域。全方位扩大区域合作，积极联动周边，推进皖南国际旅游文化示范区规划编制和政策争取；加快融入长三角城市群和杭州都市圈，在参与泛长三角区域发展分工合作上取得新进展。全力对接世博会，办好“安徽周·黄山日”活动，做好旅游、招商及名优特产品展示推介和“网上世博会”宣传工作。积极组团参加徽商大会、中博会、厦门投洽会等知名展会。加强对外友好交往，做好外事侨务和台湾事务工作。确保黄山口岸扩大对外开放通过国家验收，正式开通定期国际航班。
　　增强县域发展动力和活力，壮大综合经济实力。进一步突出县域在全市经济发展中的主体地位，强化县域、镇（乡）域经济考核体系导向作用，落实全面扩大县级管理权限各项政策，大力推进11个省级扩权强镇试点镇建设，强化市直部门服务、指导和协调职责，全力争取省里在项目建设、资金安排、用地指标等方面的支持，确保在发展速度上，试点镇高于县域、县域高于全市。鼓励全民创业，优化民营企业发展环境，培植更多更强的市场主体，提高县域工业化水平。加快推进城乡统筹发展，促进城镇化与新农村建设良性互动，着力提升城镇化水平。建立健全有利于农民市民化的体制机制，引导有条件的农民在城镇就业和落户。以城乡结合部和景区景点周边农村为重点，加快城镇基础设施向农村延伸，率先实现城乡一体化。
　　（九）不断改善民计民生，维护社会和谐稳定
　　坚持以人为本，大力推进民生改善工程，认真落实构建公共服务设施体系和建设平安和谐优秀区实施意见，让人民群众在发展中得到更多实惠，让社会在发展中更加和谐。积极做好民生工作。加大力度、创新方式，实施好35项省、市民生工程，力争走在全省前列。实施更加积极的就业政策，统筹做好高校毕业生、农民工、城镇困难群众等重点人群就业工作，确保新增城镇就业1.67万人、就业困难人员再就业2100人，农民工技能培训1万人。不断完善社会保障体系，以非公经济组织、灵活就业人员和农民工为重点，加大社会保险扩面征缴力度，全市城镇基本养老、医疗、失业、工伤、生育保险参保人数分别达到11.3万、32万、10.7万、8万和5.5万人，做好工伤保险、城镇居民基本医疗保险市级统筹和企业职工基本养老保险省级统筹工作。开展新型农村养老保险试点。提高城乡低保标准，提高企业退休人员基本养老金和部分优抚对象待遇水平。建立五保供养水平自然增长机制，推进光荣院和社会（儿童）福利中心建设。继续实施精神病患者防治项目，完善城乡大病医疗救助制度，新型农村合作医疗筹资标准提高到150元，参合率保持在94%以上。加大廉租住房等保障性住房建设力度，改善城乡低收入家庭居住条件。加强蔬菜副食品流通体系建设和市场监管，新建“菜篮子”基地4000亩，努力提高蔬菜自给率，保持价格基本稳定。完成第一轮116个贫困村整村推进工程，实施好产业化扶贫和“雨露计划”，力争7000人稳定脱贫。
　　积极发展社会事业。巩固提高义务教育发展水平，推进公共义务教育资源均衡配置，进一步改善办学条件，完成中小学校舍安全工程60%的改造建设任务。加快普及高中阶段教育。大力发展职业教育，加快市职业技术学院和民航职业技术学院建设，对中等职业学校农村家庭经济困难学生和涉农专业学生免学费。提高电大现代远程教育质量，支持黄山学院加快应用型本科院校建设。深入实施人才强市战略推进工程，不断优化人才培养、引进和使用环境，进一步发挥黄山籍旅外人士的重要作用。完善公共卫生服务体系，加快城乡基层医疗卫生服务机构标准化建设，深入开展医疗质量万里行和医院管理年活动，提高医疗服务和妇幼保健水平，增强甲型H1N1流感等重大传染病防控能力。弘扬新安医学，发展中医药事业。加强农村药品供应网和监督网建设，加大食品药品安全监管力度。广泛开展爱国卫生运动，积极争创国家卫生城市。努力稳定全市低生育水平，加强流动人口管理服务工作，完善计划生育家庭奖励扶助和特别扶助制度，开展“村为主”合格村和示范乡镇创建活动，力争所有区县和90%以上的乡镇计生服务机构达标。抓好农村留守儿童之家建设，积极完善老年人和青少年活动设施。认真做好第六次全国人口普查工作。
　　积极抓好社会管理。推进和谐社区建设，完善村务公开和民主管理长效机制，提高城市社区“四有”和信息化建设水平。支持工会、共青团、妇联等人民团体参与社会管理及公共服务。做好新形势下的民族宗教工作。推进军民融合式发展，加强国防教育、国防动员和民兵预备役及人防建设，落实优抚安置政策，巩固发展双拥创建成果。全面落实安全生产责任制，构建“8+1”长效机制，推进基层安全生产标准化百户示范点创建工作，力保安全生产10项指标控制在省下达范围之内。切实做好地质灾害防治和气象、地震等防灾减灾工作。加强应急预案完善、应急平台建设和应急力量整合，提升应对处置突发公共事件能力。深入开展“五五”普法和“江淮普法行”活动，拓展法律援助和法律服务。强化信访工作责任制，加强人民调解工作，及时排查调处矛盾纠纷，积极防范群体性事件。夯实社会治安综合治理基层基础，推进公安“三基”工程及“三项建设”，进一步完善治安防控体系，有效预防和严厉打击违法犯罪活动，确保社会和谐稳定、人民安居乐业。

　　各位代表！加快转变经济发展方式，促进经济社会平稳较快发展，对政府工作提出了新的更高要求。我们一定要切实加强政府自身建设，不断优化政治生态，做到“人人有激情，时时在状态”，努力建设一个不辜负党的希望和人民重托的政府。
　　一要振奋精神，真抓实干。面对金融危机的冲击、区域之间的竞争以及与先进发达地区的差距，我们深感压力巨大、责任重大。必须进一步牢固树立科学发展理念，强化发展第一要务意识，增强时不我待的紧迫感，保持勇争一流、奋发有为的精神状态，一心一意抓发展、聚精会神搞建设。要不断学习、善于学习，深入调研、系统调研，树立建设现代国际旅游城市的世界眼光和战略思维，增强攻坚克难、推进落实的工作本领，善于从宏观形势上把握利用，从外地发展上借鉴结合，从基层首创中总结推广，从传统继承中创新变革，把重点工作筹划得更有特色、推进得更有水平，不断增强政府工作的创造力。要弘扬求真务实的作风，落实行政首长负责制、工作责任制和责任追究制，减少不必要的应酬和开会发文，腾出时间和精力，一线推动、一抓到底，敢于动真、敢于碰硬，努力形成层层衔接、环环相扣、事事落实的工作格局，切实增强政府工作的执行力。
　　二要强化效能，提升服务。人民政府为人民，人民公仆必须尽心尽力为群众服务、为基层服务，尽职尽责为企业服务、为发展服务。要巩固第二批学习实践科学发展观活动成果，继续推进突出问题整改；扎实开展第三批学习实践活动，切实转变基层单位作风和行风。要坚持不懈抓好机关效能建设，结合市县政府机构改革，进一步健全部门牵头协调配合机制，深化行政审批制度改革，加强市县（区）两级行政服务中心建设，完善项目联合审批、基本建设收费“一表清”等制度，积极发展电子政务，提高办事效率和服务水平。要大力推进政务公开，进一步落实政府信息公开条例和新闻发布制度；对出台的政策、议定的事项要说到做到、说好做好、践行践诺、实干实效，切实提高政府工作的透明度和公信力。要继续推进“四大整治”，办好“政风行风热线”和“市民连线”，深入开展效能、政风和行风“三位一体”评议以及“百家企业评机关”活动，综合运用目标管理、任务分解、考核督查、投诉问责、行政监察、明查暗访等手段，有力解决有令不行、有禁不止现象和慢作为、不作为、乱作为问题。
　　三要依法行政，树好形象。切实把依法行政作为政府工作的行为准则，健全行政执法体制，加强行政执法协调，规范自由裁量等行为，落实执法责任制和过错责任追究制，做好行政复议、应诉和赔偿等工作，促进严格执法、公正执法、文明执法。贯彻民主集中制原则，严格执行重大行政决策程序规定，推行咨询、公示、听证、论证、评估和网络问政等制度，推进决策的科学化、民主化和法制化。自觉接受人大依法监督和政协民主监督，办好人大代表议案、建议和政协提案，经常向人大报告、向政协通报重大事项，认真落实人大审议的决议决定，注重采纳政协的调研成果和意见建议。广泛听取民主党派、工商联、无党派人士和各人民团体的意见，主动接受社会公众和新闻舆论监督。全面落实党风廉政建设责任制，更加扎实地推进惩防体系建设。建立统一的招投标和政府采购平台，专项治理工程建设领域突出问题，强化经济责任和政府投资项目审计，继续纠正损害群众利益的不正之风，严肃查处各类违法违纪案件，努力从体制机制上铲除腐败滋生的土壤，确保人民赋予的权力始终为人民谋利益，始终在阳光下运行。

　　各位代表!新的一年，孕育新的希望；新的征程，召唤新的奋斗！让我们在市委的坚强领导下，进一步解放思想，开拓创新，为全面完成和超额完成今年各项目标任务而团结拼搏，为加快建设现代国际旅游城市、谱写黄山人民美好生活新篇章而不懈努力
